# Preimplantation genetic testing for Huntington disease: the perspective of one Portuguese center

**DOI:** 10.1097/j.pbj.0000000000000048

**Published:** 2019-09-04

**Authors:** Diogo Ferreira, Berta Carvalho, Ana P. Neto, Joaquina Silva, Ana M. Póvoa, Alberto Barros, Filipa Carvalho

**Affiliations:** a Serviço de Genética, Departamento de Patologia, Faculdade de Medicina, University of Porto; b Instituto de Investigação e Inovação em Saúde, i3s, Universidade do Porto; c Centro de Genética da Reprodução A. Barros; d Departamento de Ginecologia-Obstetrícia e Pediatria, Faculdade de Medicina, Serviço de Ginecologia e Obstétricia, Centro Hospitalar Universitário S. João, Universidade do Porto, Porto, Portugal.

**Keywords:** assisted reproductive technologies, embryo, Huntington, preimplantation genetic testing

## Abstract

**Background:**

Huntington disease (HD) is an autosomal dominant late-onset neurodegenerative disease caused by an unstable cytosine-adenine-guanine trinucleotide repeat expansion in the huntingtin (*HTT*) gene. Preimplantation genetic testing (PGT) is a diagnostic procedure available for these individuals, because they carry a high risk of transmitting this genetic condition to their offspring.

**Methods:**

Information about 15 HD couples referred for PGT and 21 cycles performed from 2009 to 2018 was collected retrospectively. PGT provide direct testing of embryos obtained after intracytoplasmic sperm injection, using polymerase chain reaction multiplex as the genetic testing protocol.

**Results:**

PGT for HD was performed in 15 couples, with no history of previous attempts, in a total of 21 cycles. The mean number of biopsied embryos per cycle was 4.9. The amplification efficiency in blastomeres was 87.4%. From the 90 amplified embryos, 32 were normal and suitable for transfer. The mean number of transferred embryos per couple was 1.2.

Overall, 3 positive human chorionic gonadotropin tests were obtained in 3 couples, resulting in 2 clinical pregnancies. The 2 ongoing clinical pregnancies had normal evolution, and culminated in 2 deliveries, resulting in the birth of 2 healthy children.

**Conclusions:**

PGT for HD is considered an effective and safe reproductive option for couples who are at risk of transmitting HD, when proper genetic and reproductive counseling is warranted.

## Introduction

Huntington disease (HD) is an autosomal dominantly inherited, late-onset neurodegenerative disease caused by a dynamic mutation in the huntingtin (*HTT*) gene: an expanded cytosine-adenine-guanine (CAG) triplet repeat.^1^*HTT* gene is responsible for the synthesis of the huntingtin protein. Normally, the CAG segment is repeated 10 to 35 times within the gene. In patients with HD the CAG segment is repeated 36 to >120 times. HD is observed at reduced penetrance for repeat ranging between 36 and 39 and at full penetrance for repeat counts >40.^2^ Repeat length is not stable during meiosis, and it can expand in the subsequent generations, particularly when mutation is paternally derived.

Normal HTT plays a vital role in brain development, being mainly found in striatum and cerebral cortex. Mutated HTT has larger dimensions, due to a polyglutamine repeat in its structure. The elongated protein is fragmented in smaller toxic portions, which attach to each other and accumulate in different tissues, mainly in neurons, inducing their dysfunction and, ultimately, their death.

HD prevalence in Western Europe varies between 5 and 10 per 100,000, similar to what is observed in Portugal.^3,4^

Clinically, symptoms begin at age of 35 to 44 years and rapidly progress, significantly affecting patients’ quality of life. CAG repeat length is inversely correlated to age of onset. In fact, juvenile HD, a variant form of HD in young adults, is characterized by a large number of repeats, usually >60. Overall survival stands at 15 to 18 years and there is no current effective treatment.^3^

Preimplantation genetic testing (PGT) is performed for couples at a high risk of transmitting a known genetic condition to their offspring and allows the diagnosis of chromosomal structural rearrangements (PGT-SR) and monogenic diseases (PGT-M). PGT-M is one of the reproductive options available for these individuals, because there is a 50% risk of a carrier transmitting the mutation to the offspring.^4^ It requires a multidisciplinary approach by a team of experts in gynecology/obstetrics, embryology, and medical genetics, which will follow the couple from the adequate genetic counseling until the birth of a healthy child.

PGT-M is an alternative to prenatal diagnosis, involving the biopsy and genetic testing of single or few cells from preimplantation embryos and transfer of unaffected embryos for the genetic condition being tested to the patient's uterus. PGT-M avoids the risk of induced abortion, the psychological burden associated to termination of pregnancy, and it is the most suitable option for couples with an increased genetic risk combined with infertility.

Despite its numerous advantages, this procedure presents some risks and ethical and legal issues. It is technically complex and misdiagnosis may occur due to allele drop-out (ADO), an event in which one of the alleles is not properly amplified, and mosaicism, in case the biopsied blastomere is not representative of the total embryo. Main ethical problems relate to the moral status of the human embryo, embryo manipulation via assisted reproductive techniques, and eugenics.^5^

The Department of Genetics in the Faculty of Medicine/Centro Hospitalar Universitário São João has been the only Portuguese public center, since 1998, performing this technique. Since then, the range of chromosomal disorders and monogenetic disorders for which PGT is available has expanded worldwide. In 2009, for the first time, it was performed PGT-M in an HD couple in Portugal.

The main goal of this work was to provide an overview about the uptake and outcome of PGT-M technique in HD couples, in the perspective of a Portuguese public center.

## Methods

All therapeutic procedures were done in accordance with the National Ethical Committee and National Council for Assisted Medical Reproduction. Informed consent was obtained from both partners after careful explanation of the treatment technique. This study was approved by the Ethical Committee from Centro Hospitalar S. João (Protocol n° 357/18).

Couples obtained genetic and reproductive counseling by a clinical geneticist before being referred for PGT-M. All patients had normal karyotypes and were considered suitable candidates for this procedure.

Controlled ovarian hyperstimulation was done by a gonadotropin releasing hormone agonist or antagonist protocol on female patients. After this treatment, oocytes were collected by ultrasonography-guided follicular aspiration. Oocytes were fertilized by intracytoplasmic sperm injection (ICSI), which is preferred to conventional in vitro fertilization, because it prevents DNA contamination with sperm and/or cumulus cells during embryo biopsy. Embryo's development was carefully evaluated every day.

Embryos of type A (no anucleated fragmentation), type B (1%–20% fragmentation), and type C (21%–50% fragmentation) were biopsied in day 3 after ICSI. One (embryos with 6 cells) or 2 (embryos with 7 or more cells) blastomeres were removed from each embryo.

There are different strategies to perform genetic analysis on single cells, but the most widely procedure is a multiplex polymerase chain reaction (PCR). In this protocol, amplification of the disease-associated locus along with different informative polymorphic markers, known as “short tandem repeats,” which flank the mutated *HTT* gene is done (D4S1614, D4S412, D4S127, and IVS-1 intronic marker). This strategy overcomes the potential threat of ADO and allows the detection of contamination.

Before PCR amplification, blastomeres were lysed with proteinase K by incubation at 45°C for 15 minutes and then 98°C for 10 minutes. Five sets of primers labelled with different fluorochromes were used in multiplex PCR (Platinum Multiplex PCR Master Mix, Applied Biosystems, Foster City, Califórnia, USA) with the following PCR conditions: initial denaturation at 95°C for 2 minutes and then 45 cycles with 95°C for 30 seconds, 60°C for 90 seconds, and 72°C for 60 seconds. Final extension was done at 60°C for 30 minutes. The fluorescent PCR products were separated based on their size and fluorochrome in an ABI PRISM 3500 Genetic Analyzer and analyzed using the appropriate software (Genemapper, Applied Biosystems).

After genetic diagnosis, 1 or 2 unaffected embryos (without expansion) and with good morphological quality were selected and transferred into the uterus, on blastocyst stage (day 5 post-ICSI).

## Results

From 2009 to 2018, fifteen HD couples were referred for PGT-M for HD. Twenty-one cycles were performed and 6 couples repeated the cycle once corresponding to a mean of 1.4 cycles per couple. Mutation was paternally derived in 6 couples, whereas in the other nine couples it was maternally derived corresponding to a male:female ratio of carriers/at risk persons of 1:1.5. None of these couples had a previous PGT-M attempt. The mean female age at beginning of each cycle was 34.5 years (Table 1).

**Table 1:**
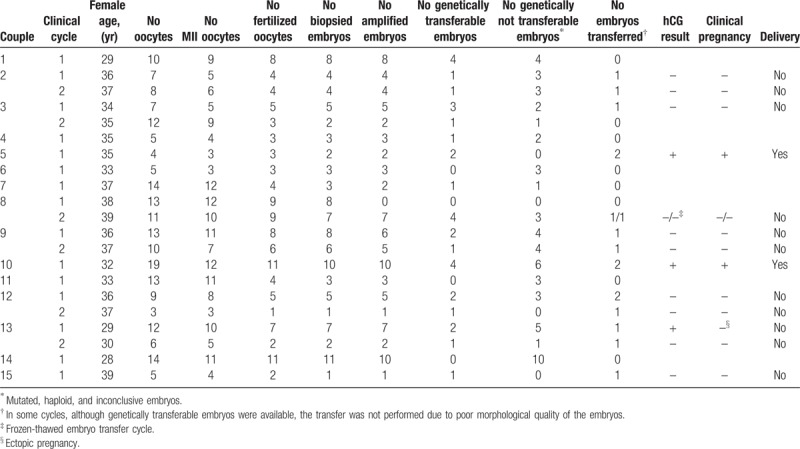
Preimplantation Genetic Testing-M cycles performed for Huntington disease couples

One couple had a reported male infertility history and so the sperm was obtained by Testicular Sperm Extraction.

Following ovarian stimulation, the mean number of cumulus-oocyte complexes retrieved per cycle was 9.5 (ranging from 3 to 19). Overall, a mean of 7.6 oocytes per cycle were considered mature (metaphase II oocytes) and a mean of 5.6 oocytes were successfully fertilized (Table 1).

The mean number of biopsied embryos per cycle was 4.9. Laser drilling was the preferred method for zona breaching, during embryo biopsy. Embryos were biopsied at cleavage-stage, on day 3, and 1 or 2 blastomeres were retrieved for genetic analysis.

**Figure 1:**
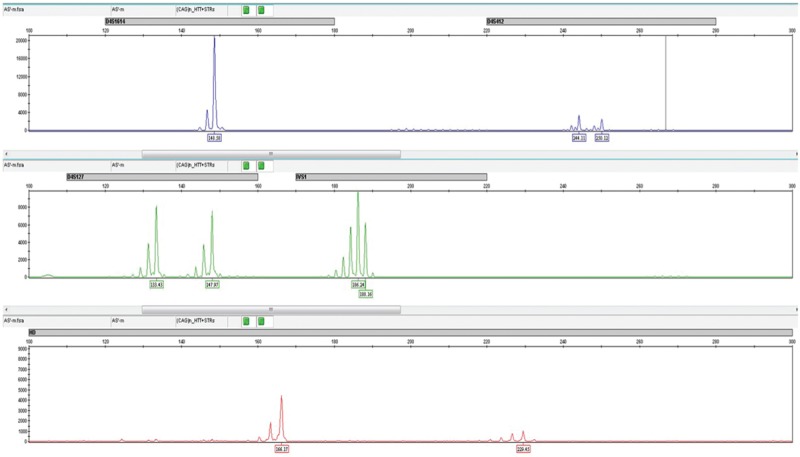
Electropherogram of the multiplex PCR amplifying simultaneously the cytosine-adenine-guanine (CAG) repeat (red) and the polymorphic markers D4S1614 (blue), D4S412 (blue), D4S127 (green), and IVS1-intronic marker (green).

Multiplex PCR was the genetic testing method used for DNA amplification of each biopsied embryo. In average, 4.3 embryos per PGT-M cycle obtained a positive signal in the PCR reaction (Fig. 1). The amplification efficiency in blastomeres was 87.4%. From the 90 amplified embryos, 32 were genetically transferable, whereas 58 were genetically not transferable (including mutated, haploid, and inconclusive embryos) (Table 1). ADO was detected in 3 embryos, from 3 different cycles.

In 13 cycles it was possible to perform embryo transfer, with 17 embryos being transferred (including a frozen-thawed embryo transfer cycle from couple 8), corresponding to 14 transfers and a mean number of transferred embryos per couple of 1.2 (in the range 1–2). In some couples, although genetically transferable embryos were available, the transfer was not performed due to poor morphological quality of the embryos.

Overall, 3 positive human chorionic gonadotropin (hCG) tests were obtained in 3 couples, resulting in 2 clinical pregnancies. One of the positive hCG ended as an ectopic pregnancy and termination of pregnancy (couple 13). The clinical pregnancy rate was 14.3% per transfer. Amniocentesis confirmed PGT-M result in 1 couple, whereas the other couple decided not to perform prenatal diagnosis.

The 2 ongoing clinical pregnancies had normal evolution, and culminated in 2 deliveries (delivery rate/embryo transfer of 14.3%). The 2 cesarean sections deliveries resulted in the birth of 2 healthy children, 1 girl and 1 boy (Table 2).

**Table 2:**
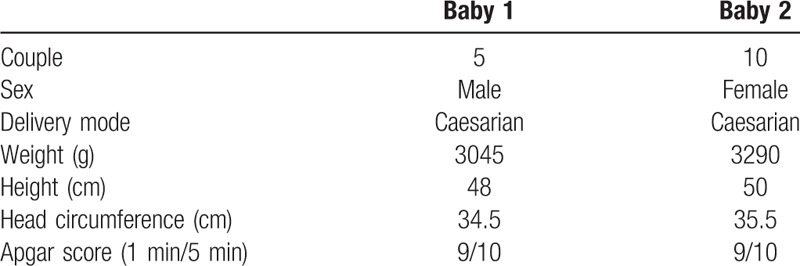
Clinical information of the 2 babies at birth

## Discussion

Reproductive options available for couples with genetic disorders should be analyzed in each individual case, concerning their advantages and disadvantages. Although prenatal diagnosis has higher success rates, the possibility of termination of pregnancy in case of an unfavorable result represents one of the major concerns. Whether the introduction of PGT for HD has reduced or not the use of prenatal diagnosis in these patients is an interesting question, and should be studied in further investigations in our population.

PGT has specific indications and its implementation is regulated by law and subject to the National Council for Medically Assisted Procreation approval. One of the indications accepted by law is HD. PGT is one of the available reproductive options for these couples, acknowledging the possibility of analysis for the presence of the triplet expansion, and/or genetically linked markers associated with the dynamic mutation, in human embryos.

This analysis can be performed in 2 different modalities: direct testing of embryos or exclusion testing. The latter is not approved in Portugal, but it is performed in other European centers, when couple decided not to be informed about their HD carrier status, and do not want to be subjected to presymptomatic testing.^6^

European Society of Human Reproduction and Embryology PGT Consortium, established in 1997, collects, retrospectively and prospectively, data on PGT cycles, pregnancies, deliveries, and children. The later published report, covering monogenetic diseases, HLA typing, and chromosome abnormalities, documented cycles performed from 2011 to 2012.

Our main results on PGT cycles for HD patients were compared to those internationally published, although we were aware on implications of working on different sample sizes.

The mean age of woman at beginning of the first cycle (34.5 years) was a bit higher than that reported in literature (32 years).^7^ This may be caused by the delay of Portuguese couples on searching for these treatments, waiting list, or by the lack of available information about PGT.

In our study, male:female ratio of HD carriers or at-risk persons was 2:3, matching the 40:60 ratio reported for couples opting for presymptomatic testing.^3^

Although all couples were selected for ICSI procedure, most parents who undergo PGT do not have fertility problems, except 1 couple with a reported male infertility history. None of the 15 couples have benefited on this technique in the past.

According to literature, day 3 cleavage stage embryo biopsy remains the preferable biopsy method for PGT-M cycles, although it may switch to day 5 biopsy in the very near future.^7^ In our study, 100% of embryos were biopsied in day 3 after ICSI.

From the embryos successfully biopsed, 87.4% gave a diagnostic result (vs 91%, from literature).^7^ Multiplex PCR may be subjected to several problems, including sample contamination, total PCR failure, and ADO. The latter phenomenon was detected in 3 (3.3%) embryos, from 3 different cycles. Increasing number of linked informative polymorphic markers are being used, to reduce the risk of misdiagnosis.

The mean number of transferred embryos per couple was 1.2 (ranging from 1 to 2), comparable with that found in the literature.^8^

The clinical pregnancy rate and the delivery rate were both 14.3% per embryo transfer, which significantly differs from what is observed in our center for other pathologies and also depicted in literature.^7^ One potential reason, which may explain this difference, is the small sized PGT-M sample for HD in this study.

The pregnancy rates of the Department of Genetics in the Faculty of Medicine/Centro Hospitalar Universitário São João are in accordance with international data on PGT pregnancy rates for other pathologies.

Despite the reported differences, we concluded that PGT-M for HD is considered an effective and safe reproductive option for couples who are at risk of transmitting HD, when proper genetic and reproductive counseling is warranted.

## Acknowledgements

None.

## Conflicts of interest

The authors report no conflicts of interest.
